# Evaluating the Impact of an Iron Supplementation Program for Combating Anemia in School-Age and Adolescent Females by a Grassroots Organization in India

**DOI:** 10.7759/cureus.75608

**Published:** 2024-12-12

**Authors:** Suryansh Kataria, Samriddhi Kataria, Durga Chougule, Bindesh Bharti, Akanksha Rastogi

**Affiliations:** 1 Public Health, Delhi Public School, Gurgaon, IND; 2 Research, Freelance, Mumbai, IND; 3 Public Health, Aravindam India Foundation, Gurgaon, IND; 4 Internal Medicine, Medanta – The Medicity, Gurgaon, IND

**Keywords:** anemia prophylaxis, anti-helminthic treatment, behaviour change communication, evaluation of public health programme, grassroots organization, iron deficiency anemia, iron folic acid supplementation

## Abstract

Background and objective: Iron deficiency anemia (IDA) is a prominent cause of anemia adversely affecting the physical, mental, and social well-being of an individual. It is a major health concern and has impacted more than two billion people worldwide. It is necessary to implement programs to increase compliance rates for iron and folic acid (IFA) supplementation and educate individuals about anemia. In this study, we evaluate the impact of a non-profit organization's IFA supplementation program for school-age and adolescent females to combat anemia.

Methodology: This study was conducted via a grassroots organization that used a peer network to integrate with the existing national anemia campaign in school-age and adolescent females. This single-arm preintervention and postintervention study conducted over four months from June to September 2023 evaluated the effect of a one-time anti-helminthic treatment and weekly IFA supplementation (45 mg elemental iron and 0.4 mg folic acid per tablet for seven to nine-year-old females; 60 mg elemental iron and 0.5 mg folic acid per tablet for 10 to 19-year-old females) for 90 days. Hemoglobin levels at baseline and post-IFA supplementation were assessed using point-of-care direct reading hemoglobinometers. The pre- and post-test hemoglobin values were compared by a paired t-test.

Results: A total of 160 females visiting the foundation were approached, out of which 146 agreed to participate in the study. The baseline hemoglobin levels among 146 females were 10.9 ± 1.7 g/dL, and 103 (70.5%) of them were observed to have anemia. Due to a loss of follow-up, 99 participants were evaluated for pre- and post-IFA supplementation hemoglobin levels. Among 99 females, anemia was observed in 68.7% at baseline. The baseline hemoglobin level of 11.1 ± 1.7 g/dL significantly raised to 11.7 ± 1.4 g/dL after IFA supplementation (p<0.001). After IFA supplementation, the baseline distribution of participants with normal hemoglobin rose to 47.5% (post-test) from 31.3% (pre-test), showing a difference of 16.2%. The normal hemoglobin level distribution across the age groups after the weekly IFA supplementation program improved by 17.2% in participants aged seven to 10 years (from 31.4% to 48.6%), 17.7% in 11 to 14 years (from 29.45% to 47.1%), and 7.7% in those aged 15 to 19 years (from 38.5% to 46.2%).

Conclusion: Grassroots organizations can be pivotal in enhancing the efficacy of IFA supplementation programs, particularly in combating anemia among children. Behavior change communication and peer networks of such organizations can be integrated into existing programs to significantly contribute to improving the compliance rate of IFA supplementation programs.

## Introduction

Anemia is a condition characterized by insufficient RBCs or hemoglobin, hindering the adequate supply of oxygen for physiological demands, and thereby impacting physical, mental, and social well-being [1]. Malnutrition, genetic disorders, infectious diseases, and physiological conditions like menstruation and pregnancy contribute to an anemic condition [2]. Anemia is a significant global health issue, particularly in low- and middle-income countries, with the WHO 2023 fact sheet underscoring that 40% of children (aged six to 59 months), 37% of pregnant women, and 30% of women of reproductive age are anemic [3].

Iron deficiency (ID), a major cause of anemia, impairs oxygen transport, DNA regulation, and muscle metabolism, negatively impacting academic performance, work productivity, and overall well-being [4]. Insufficient dietary iron intake, impaired iron absorption, chronic blood loss, and abnormal iron metabolism contribute to iron deficiency anemia (IDA) [5]. Women of reproductive age are vulnerable to IDA as they experience higher iron requirements due to menstrual blood loss and increased iron demands during pregnancy to support fetal growth [6]. Iron deficiency progresses from depletion of iron stores to iron-deficiency erythropoiesis that is characterized by reduced iron stores but normal hemoglobin levels, and finally to IDA, with low hemoglobin, hematocrit (the proportion of RBCs in blood by volume), and mean corpuscular volume (a measure of erythrocyte size) [7]. The WHO guidance released in 2020 reports IDA in 33% of non-pregnant women, 40% of pregnant women, and 42% of children [8]. A nationwide Comprehensive National Nutrition Survey 2016-2018 (CNNS 2016-18) conducted on adolescents aged 10 to 19 years reported a 12% prevalence of IDA. The survey also identified ID as a strong predictor of anemia among adolescents [9]. Iron deficiency anemia is a preventable and treatable condition linked to poverty, inadequate care, and management. Incorporation of iron-rich diets, supplementation, and fortification strategies can effectively reduce the risk of anemia, increase hemoglobin concentration, and improve mental development [10].

Combating anemia for maternal, infant, and young child nutrition is a major goal of the World Health Assembly Global Nutrition Targets, the United Nations 2030 Agenda for Sustainable Development, and the WHO. Achieving this goal requires the development of a comprehensive framework and collaborative efforts from various stakeholders to implement strategies for executing the framework in various countries [3]. Currently, the WHO global guidelines for the prevention of IDA in public health promote intermittent (weekly) iron supplementation, home fortification with multiple micronutrient powders, dietary plan modification, and periodic deworming for preschool and school-going children, adolescent girls, and women. [11]. The Government of India launched Anaemia Mukt Bharat in 2018 to target reduction in anemia among women, children, and adolescents through a prophylactic iron and folic acid (IFA) supplementation program and intensified year-round behavior change communication (BCC). The campaign also included testing of anemia using digital methods, periodic deworming, provision of point-of-care treatment, addressing non-nutritional causes of anemia, training medical officers, and increasing field-level awareness among auxiliary nurse midwives (ANMs), Anganwadi workers (AWWs), and accredited social health activists (ASHAs) [11]. The point-of-care treatment provided was in reference to the guidelines for control of IDA, which recommended a weekly dosage of 45 mg elemental iron and 0.4 mg folic acid per tablet for 61 months for those aged 10 years; 60 mg elemental iron and 0.5 mg folic acid per tablet for 10- to 19-year-olds; and 100 mg elemental iron and 0.5 mg folic acid per tablet for women in the reproductive age group [12].

Socioeconomic disadvantages, recurrent lack of access to unreached areas, limited community education, and absence of community participation were the prominent barriers affecting the implementation of iron supplementation programs. Therefore, a multi-pronged approach prioritizing proactive interventions and establishing community-level nutritional monitoring programs for children and adolescents may be effective for anemia prevention [13-15]. The challenges were addressed through the involvement of community health worker-led interventions to educate and counsel parents to improve children’s anemia cure rate [16,17]. We involved a peer group to screen school-age and adolescent females for signs of anemia through opportunistic screening at grassroots non-profit organizations involved in conducting academic and extra-curricular activities to ensure adherence to IFA supplementation among this demographic through daily interactions. Here, we investigate the impact of grassroots non-profit organizations in implementing weekly iron supplementation among school-age and adolescent females in treating anemia through peer networks.

## Materials and methods

Study design and setting - This single-arm pre- and post-intervention study was conducted at Aravindam Foundation, a non-governmental organization (NGO) in Gurugram, Haryana. This study was done under the Anaemia Mukt Haryana (AMH) initiative launched by the National Health Mission, Haryana over four months from June to September 2023. The study was approved by the Medanta Institutional Ethics Committee (1457/2022). The ethics committee approved a note dated 24 June 2023 permitting the conduct of anemia screening in NGOs, schools, and colleges of Delhi NCR. Written consent was obtained from the participant’s parents/guardians before recruiting them for the study. The participants voluntarily agreed to participate in the study. 

Sample size

Studies have reported a 70% anemia level among children from India. [18] It was assumed that there would be a decline of 15% in the anemia level with the proposed intervention. Therefore, the following formula has been used: n=((Z1-α/2+Z1-β)/ES)2 where ES=(p1-p0)/√(p1(1-p1)); p1 denotes the baseline value of parameter (anemia level), p0 denotes the end line value of parameter (anemia level); Zα is the value of standard normal variate corresponding to α level of significance; Zβ is the standard normal deviate for desired power effect size (ES). The assumptions considered were p1 = 0.70, p0 = 0.55, Zα = 1.96, and Zβ = 1.282. The minimum sample size calculated was 98.

Population and inclusion and exclusion criteria

The study participants were girls in the defined age group visiting the organization for academic learning. They volunteered to participate in the study. Females between seven and 19 years of age were included in this study. Participants already on IFA supplements were excluded from the study. 

Intervention

Per the guidance note draft on 'Anemia Elimination Week' under AMH, all study participants irrespective of their hemoglobin concentration were administered prophylactic IFA tablets (45 mg elemental iron and 0.4 mg folic acid per tablet for seven to nine-year-old females; and 60 mg elemental iron and 0.5 mg folic acid per tablet for 10 to 19-year-old females) once per week for 90 days and a single dose of anti-helminthic treatment (tablet albendazole 400 mg). Mild or moderate anemia detected in seven to nine-year-old female children was managed by administration of 3 mg iron/kg/day for two months. Conversely, adolescent females detected with mild or moderate anemia were recommended an intake of two IFA tablets daily for three months. Females with severe anemia were urgently referred to district hospitals for standard-of-care treatment until their hemoglobin levels were normal. The study team comprising of self-volunteers, clinicians, and the foundation manager ensured program management, supply of supplements, peer coordination, and record maintenance. The academic and extracurricular activities by the organization were held in open spaces where the study team regularly interacted with the participants and ensured the continuation of prophylaxis (behavior change communication and monitoring through peer networks). Well-being among participants was maintained through the provision of healthy meals, the arrangement of a food *mela *(festival), and fun activities.

Data collection

A total of 160 females were approached among which 146 agreed to participate in the study. There was a loss to follow-up due to which only 99 participants completed the pre-test and post-test. Demographic details (age, weight, and height) and hemoglobin levels of participants at baseline and after prophylactic management were recorded in an electronic file document which was on a password-protected computer requiring authorized access. The baseline hemoglobin levels were determined in participants and then they were categorized as mild (10.0 - 11.9 g/dL), moderate (7.0 - 9.9 g/dL), and severe (<7.0 g/dL) anemia according to the WHO classification [19]. Post-intervention hemoglobin levels were assessed after three months. The pre- and post-intervention hemoglobin levels were measured using the DiaSpect Haemoglobin Analyzer (EKF Diagnostics, Cardiff, Wales, UK). The instrument was regularly calibrated and was found to be in the admissible range provided by the manufacturer. The data quality was maintained throughout the study by a well-trained study team and the use of calibrated direct reading hemoglobinometers for measuring hemoglobin levels.

Comparison, outcomes, and statistical analysis

The pre- and post-intervention hemoglobin levels among the participants (n = 99) were compared. This study will help in understanding whether the IFA supplementation programs by grassroots-level organizations can play a pivotal role in combating anemia among children. The demographical variables (age, class, and anemia category) were represented as frequency and percentage. The pre- and post-intervention hemoglobin levels were represented as mean and standard deviation and were compared using the paired t-test. Data was analyzed on SPSS Statistics version 24 (IBM Corp., Armonk, NY, USA) and statistical significance considered at p<0.05. The data visualization was done using Microsoft Excel 2016 (Microsoft Corp., Redmond, WA, USA).

## Results

A total of 146 girls agreed to participate in the study out of which 47 (32.2%) were in the senve to 10 years age group, 77 (52.7%) were between 11 and 14 years, and 22 (15.1%) were adolescents (15 to 19 years). The average age of study participants was 11.9 ± 2.7 years (Table 1). However, some participants were lost to follow up. Therefore, post-intervention hemoglobin levels were evaluated in only 99 females. Table 1 describes the age and grade distribution of participants at baseline and follow-up.

**Table 1:** The age and grade-based frequency and percentage distribution of study participants at baseline (n = 146) and follow-up (n = 99)

Parameters	Baseline participants	Percentage	Follow-up participants	Percentage
	(n = 146)		(n = 99)	
Age (years)				
Seven to 10	47	32.2	35	35.4
11 to 14	77	52.7	51	51.5
15 to 19	22	15.1	13	13.1
Mean ±SD (range)	11.9 ± 2.7 (7-19)		11.8 ± 2.6 (7-19)	
Class				
1^st^ – 4^th^	43	29.5	32	32.3
5^th^ – 8^th^	74	50.7	49	49.5
9^th^ – 12^th^	29	19.9	18	18.2

**Figure 1:**
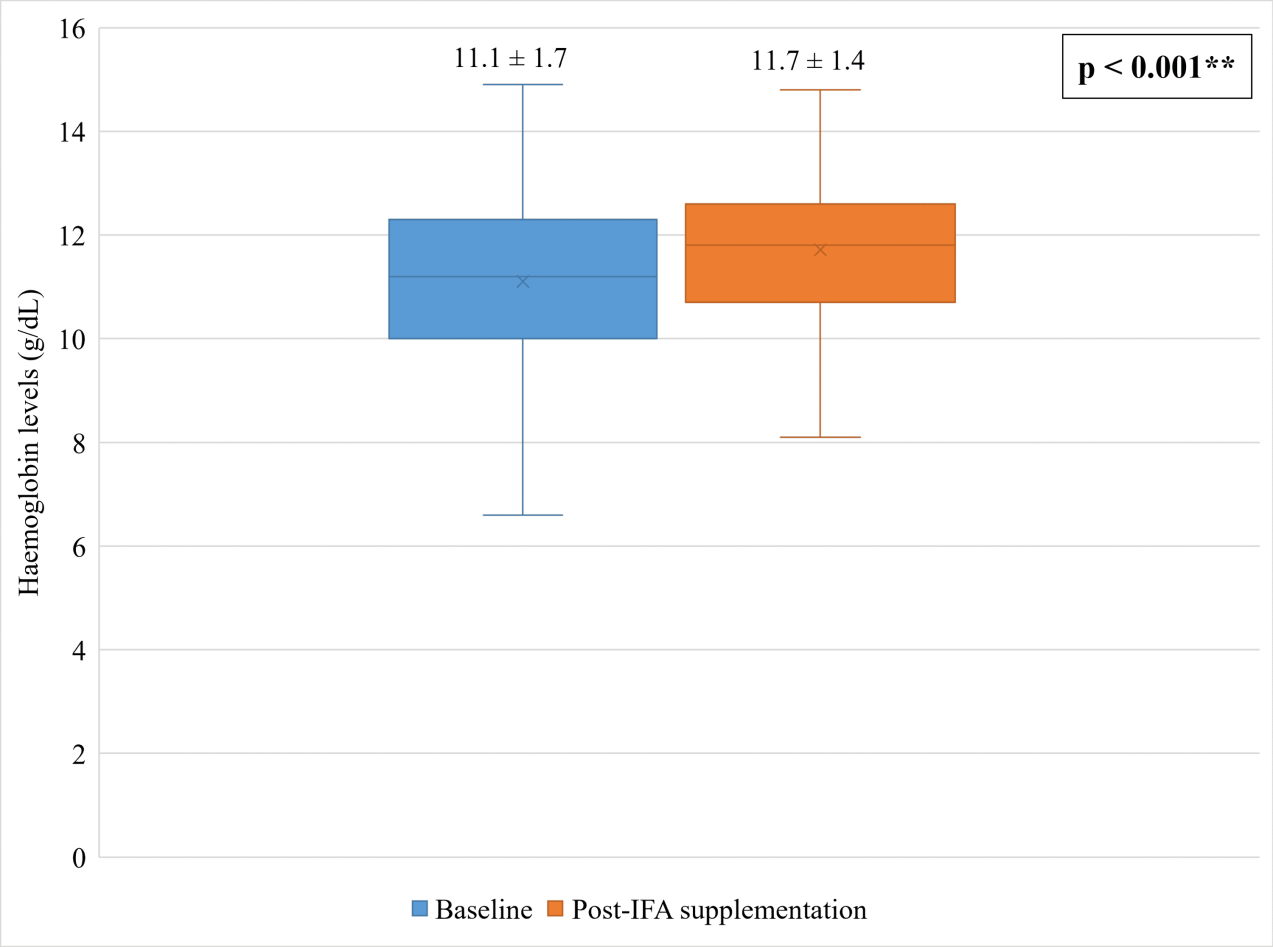
Comparison of baseline and post-IFA supplementation hemoglobin levels in participants (n = 99) The comparison between the pre- and post-test hemoglobin levels was done using a paired t-test. *A p-value <0.05 was considered statistically significant. IFA: Iron and folic acid

The average hemoglobin levels in 146 females at baseline was 10.9 ± 1.7 g/dL. Among 146 females, 103 (70.5%) were anemic with a distribution of 61 (59.2%) mild cases, 39 (37.9%) moderate cases, and three (2.9%) severe cases. Among the participants followed-up, the average hemoglobin levels improved from 11.1 ± 1.7 g/dL to 11.7 ± 1.4 g/dL after administration of IFA supplements and anti-helminthic treatment. A statistically significant mean difference of 0.6 ± 0.5 g/dL was observed in hemoglobin levels between baseline and post-IFA supplementation (p<0.001) (Figure 1).

Among the participants followed up, the distribution of hemoglobin levels improved after IFA supplementation with a higher percentage exhibiting normal hemoglobin levels (from 31.3% to 47.5%) and a lower percentage experiencing mild (40.4% from 45.5%) and moderate (12.1% from 21.2%) anemia (Table 2).

**Table 2:** Distribution of anaemia categories among participants at baseline and post-IFA supplementation (n = 99) IFA: Iron and folic acid

Anemia categories	Number of students	
	Baseline	Post-IFA supplementation
Normal (≥12.0 g/dL)	31 (31.3%)	47 (47.5%)
Mild (10.0 – 11.9 g/dL)	45 (45.5%)	40 (40.4%)
Moderate (7.0 – 9.9 g/dL)	21 (21.2%)	12 (12.1%)
Severe (<7.0 g/dL)	2 (2%)	0 (0%)

**Figure 2:**
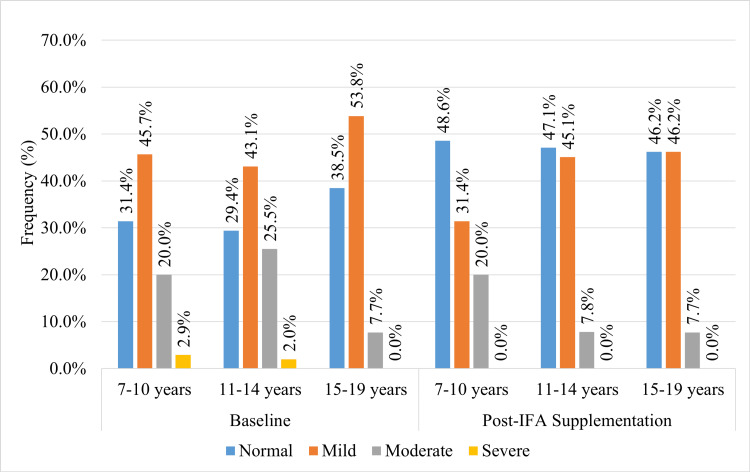
The baseline and post-IFA supplementation distribution of anaemia categories across age groups. IFA: Iron and folic acid

Figure 2 depicts the distribution of anemia categories at baseline and post-iron supplementation across the age groups. At baseline, the percentage frequency of participants having normal hemoglobin levels across the age groups was: 11/35 (31.4%) in seven to 10 years, 15/51 (29.45%) in 11 to 14 years, and 5/13 (38.5%) in 15 to 19 years. The weekly IFA supplementation program raised the normal hemoglobin level distribution to 17/35 (48.6%) in seven to 10 years (improved by 17.2%), 24/51 (47.1%) in 11 to 14 years (improved by 17.7%,) and 6/13 (46.2%) in 15 to 19 years (improved by 7.7%).

## Discussion

In this study, nearly 70.5% of the 146 female participants were detected with anemia. Of these participants, only 99 were followed up after three months of IFA supplementation. The IFA supplementation program significantly raised the hemoglobin levels among the females by 0.6 ± 0.5 g/dL (from 11.1 ± 1.7 g/dL to 11.7 ± 1.4 g/dL). Also, the distribution of normal hemoglobin was raised from 31.3% to 47.5% (a difference of 16.2%). A rise in the distribution of normal hemoglobin levels was also noted across all age groups (17.2% in seven to 10 years, 17.7% in 11 to 14 years, and 7.7% in 15 to 19 years). 

Our study reported a high prevalence of anemia among adolescent females (15 to 19 years) at 8/13 (61.5%). These findings aligned with results reported by the National Family Health Survey 5 2019-2021 (NFHS-5) in adolescent females aged between 15 to 19 years nationwide (59.1%) and in the state of Haryana (62.3%) in India [20]. However, the prevalence of anemia among adolescent females in this study was lower than that reported by NFHS-5 for the Gurugram (77.6%) district [21].

Oral iron supplementation through liquid and tablet formulation has been advocated as a cornerstone treatment to combat anemia. Administration of IFA supplementation effectively improved the hemoglobin levels from 11.1 ± 1.7 g/dL to 11.7 ± 1.4 g/dL with a mean difference of 0.6 ± 0.5 g/dL among our study participants. Post-IFA supplementation follow-up in 99 participants revealed a reduction in anemia cases. A systematic review of 55 trials reported oral or parenteral iron supplementation or dietary intake of iron-fortified milk or cereals significantly increased hemoglobin levels with a mean difference of 0.74 g/dL. The study also reported a reduction in anemia prevalence by 37.9% to 62.3% and 5.8% to 31.8% in non-malaria and malaria hyperendemic regions respectively [22].

The World Nutrition Assessment 2016, reported a high incidence of IDA in India ranking the nation at 170th position out of 180 nations. The situation was addressed by the implementation of nutrition-related programs for a budget of Rs. 36,707 crore. In addition, an extra budget of Rs. 2.07 lakh billion was set aside to boost nutrition in the population through welfare schemes [4]. Government-run programs such as Integrated Child Development Scheme (ICDS), National Nutritional Anaemia Control Program (NNACP), Weekly Iron and Folic Acid Supplementation (WIFS), and National Iron Plus Initiative (NIPI) were implemented to control anemia throughout the nation. However, these programs facilitated poor improvement in lowering the alarming magnitude of anemia. A low compliance rate of IFA at 30% was reported as a major reason for high anemia prevalence. This gap closure was done through strengthening newer strategies like social and behavior change communication (SBCC) campaigns, the initiation of a full-fledged mission such as the Anaemia Mukt Bharat initiative, involving community health centers in reaching the underprivileged regions of India, and awareness campaigns [23].

Education-based interventions on IFA supplementations and iron-fortified foods have shown to increase health literacy on anemia and reduced prevalence of anaemia in children. Community health worker interventions in parental education, counseling, and IFA supplementation programs have positively improved the hemoglobin levels in children and adolescent females with low hemoglobin levels [16,17,24]. In our study, the non-profit’s intervention in monitoring prophylaxis adherence and improvement of dietary habits among children have positively impacted a rise in normal hemoglobin distribution among study participants by 16.2%. 

Limitations

This study shows promising evidence for the inclusion of grassroots stakeholders in controlling anemia. However, it is necessary to acknowledge the limitations. The sample size was relatively small and the study design lacked a control group. The study was conducted over a short period and did not capture the long-term effects of the prophylaxis. Socioeconomic status, dietary habits, other micronutrient deficiencies, and their potential impact on iron absorption were not documented which would have strengthened the present findings. This study focuses on qualitative aspects of behavior change communication and lacks a quantitative assessment of compliance rate and participants’ behavior towards involvement of peer networking in the implementation of the IFA supplementation program. It would be essential to document pre- and post-assessments of knowledge levels and quantitative surveys using questionnaires to understand the effectiveness of improving adherence to supplementation programs.

## Conclusions

The study demonstrates the multi-pronged approach involving grassroots organizations as potential stakeholders in the implementation of IFA supplementation programs. This is done by educating children and monitoring prophylaxis adherence through behavior change communication and peer networks to combat anemia. The development of promising strategies for grassroots-level organizations will aid in addressing the critical health concern of anemia and improve the health and well-being of the anemic population.
